# Artificial cell factory design for shikimate production in *Escherichia coli*

**DOI:** 10.1093/jimb/kuab043

**Published:** 2021-07-06

**Authors:** Han-Na Lee, Seung-Yeul Seo, Hey-Jin Kim, Ji-Hoon Park, Eunhwi Park, Si-Sun Choi, Sang Joung Lee, Eung-Soo Kim

**Affiliations:** Department of Biological Sciences and Bioengineering, Inha University, Incheon 22212, Republic of Korea; STR Biotech Co., Ltd., Bioplaza 4-3, 56, Soyanggang-ro, Chuncheon-si, Gangwon-do 24232, Republic of Korea; STR Biotech Co., Ltd., Bioplaza 4-3, 56, Soyanggang-ro, Chuncheon-si, Gangwon-do 24232, Republic of Korea; Department of Biological Sciences and Bioengineering, Inha University, Incheon 22212, Republic of Korea; Department of Biological Sciences and Bioengineering, Inha University, Incheon 22212, Republic of Korea; Department of Biological Sciences and Bioengineering, Inha University, Incheon 22212, Republic of Korea; Department of Biological Sciences and Bioengineering, Inha University, Incheon 22212, Republic of Korea; STR Biotech Co., Ltd., Bioplaza 4-3, 56, Soyanggang-ro, Chuncheon-si, Gangwon-do 24232, Republic of Korea; Department of Biological Sciences and Bioengineering, Inha University, Incheon 22212, Republic of Korea

**Keywords:** Shikimate production, Cell factory design, *Escherichia coli*, Metabolic engineering, Process optimization

## Abstract

Shikimate is a key intermediate in high demand for synthesizing valuable antiviral drugs, such as the anti-influenza drug and oseltamivir (Tamiflu^®^). Microbial-based shikimate production strategies have been developed to overcome the unstable and expensive supply of shikimate derived from traditional plant extraction processes. Although shikimate biosynthesis has been reported in several engineered bacterial species, the shikimate production yield is still unsatisfactory. This study designed an *Escherichia coli* cell factory and optimized the fed-batch culture process to achieve a high titer of shikimate production. Using the previously constructed dehydroshikimate (DHS)-overproducing *E. coli* strain, two genes (*aroK* and *aroL*) responsible for converting shikimate to the next step were disrupted to facilitate shikimate accumulation. The genes with negative effects on shikimate biosynthesis, including *tyrR, ptsG*, and *pykA*, were disrupted. In contrast, several shikimate biosynthetic pathway genes, including *aroB, aroD, aroF, aroG*, and *aroE*, were overexpressed to maximize the glucose uptake and intermediate flux. The *shiA* involved in shikimate transport was disrupted, and the *tktA* involved in the accumulation of both PEP and E4P was overexpressed. The rationally designed shikimate-overproducing *E. coli* strain grown in an optimized medium produced approximately 101 g/l of shikimate in 7-l fed-batch fermentation, which is the highest level of shikimate production reported thus far. Overall, rational cell factory design and culture process optimization for microbial-based shikimate production will play a key role in complementing traditional plant-derived shikimate production processes.

## Introduction

Shikimate is a key metabolite involved in the aromatic biosynthetic pathway present in most plants and microorganisms, and an important metabolite used as a precursor for the synthesis of many valuable bioactive compounds. The metabolite is a precursor for the chemical synthesis of the neuraminidase inhibitor oseltamivir phosphate (Tamiflu®). This drug is used for the treatment of infection with diverse seasonal influenza viruses, including influenza A and B viruses, the avian influenza virus H5N1, and the human influenza virus H1N1 (Krämer et al., 2003; Dharan et al., 2009; M. Estevez & J. Estevez, 2012; Martinez et al., 2015; Diaz-Quiroz et al., 2018). Shikimate is also a key chiral starting material to many chemical substances used in the chemical and cosmic industries (Ghosh et al., 2012; Rawat et al., 2013).

**Fig. 1.**
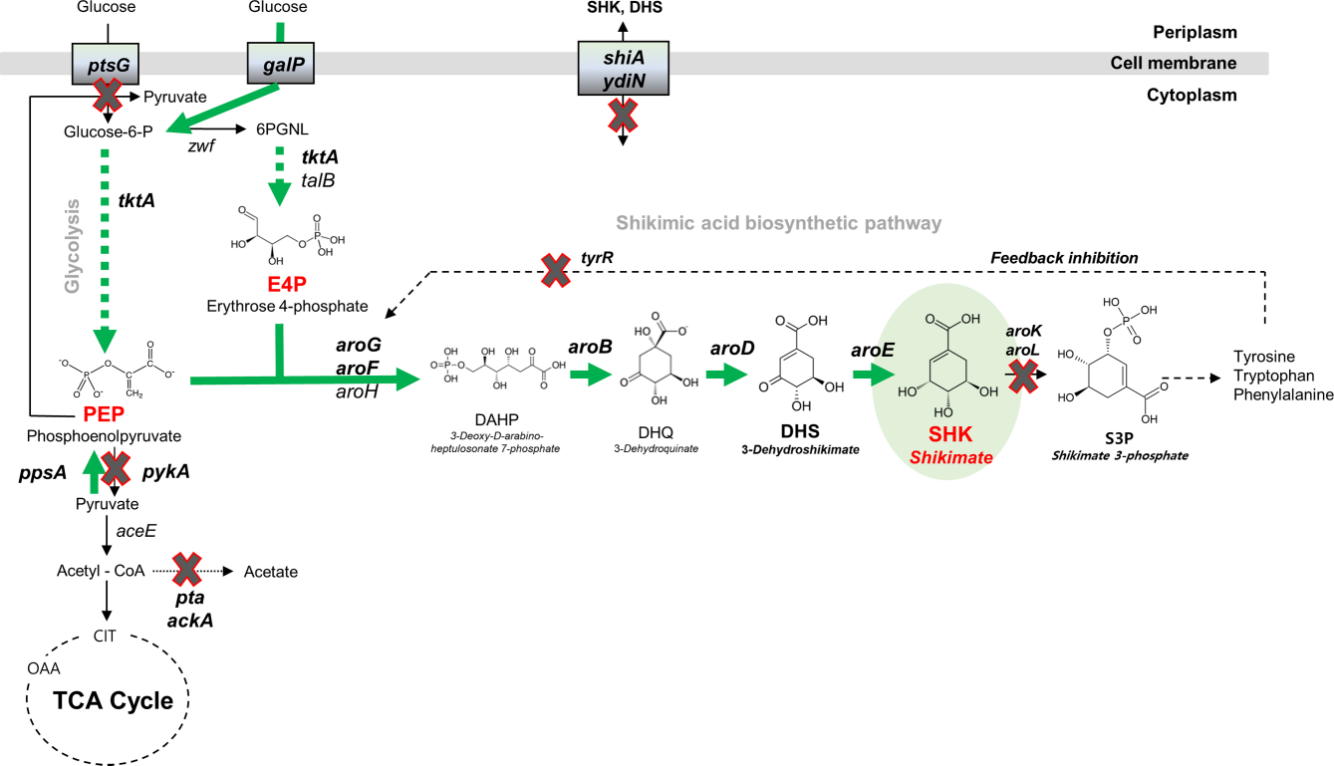
Schematic overview of metabolic pathway for SHK biosynthesis in *Escherichia coli*. Red-line crosses denote disrupted genes and bold green arrows denote steps that are overexpressed. Dashed arrows represent two or more steps. 6PGNL, 6-phosphogluconolactone; OAA, oxaloacetate; CIT, citrate; *ptsG*, glucose specific sugar: phosphoenolpyruvate phosphotransferase; *galP*, D-galactose transporter; *shiA*, shikimate transporter; *ydiN*, hypothetical transport protein; *zwf*, glucose-6-phosphate 1-dehydrogenase; *tktA*, transketolase; *talB*, transaldolase; *pykA*, pyruvate kinase 2; *ppsA*, phosphoenolpyruvate synthase; *aroG, aroF, aroH*, DAHP synthase; *aroB*, DHQ synthase; *aroD*, DHQ dehydratase; *aroE*, shikimate dehydrogenase; *aroK*, shikimate kinase; *aroL*, shikimate kinase 2; *tyrR*, tyrosine dependent transcriptional regulator.

Shikimate is typically synthesized via series of enzyme-led step-wise bioconversions shown in Fig. 1. 3-Deoxy-D-arabino-heptulosonate-7-phosphate (DAHP) is first produced through the condensation of phosphoenolpyruvate (PEP) and erythrose-4-phosphate (E4P), followed by sequential conversions to 3-dehydroquinate (DHQ), 3-dehydroshikimate (DHS), and shikimate. Shikimate is then transformed further to shikimate-3-phosphate by shikimate kinase isoenzyme I and II encoded by *aroK* and *aroL*, respectively. Shikimate-3-phosphate is then transformed to chorismate, which is finally converted to phenylalanine, tyrosine, tryptophan, and other aromatic products.

In the past few decades, the metabolic engineering approach for the overproduction of aromatic amino acids has been reported through the microbial-based shikimate biosynthesis pathway engineering (Kramer et al., 2003; Cortes-Tolalpa et al., 2014; Kruyer & Peralta-Yahya, 2017; Lee & Wendisch, 2017; Averesch & Kromer, 2018; Cao et al., 2020). Most studies optimizing the shikimate pathway focused on *Escherichia coli*. For example, several attempts have been made to solve DAHP inhibitory feedback to increase the production of aromatic compounds (Pittard & Yang, 2008; Wang et al., 2013). Several engineering strategies were also reported to reduce the consumption of the major precursors, PEP and E4P (Escalante et al., 2010; Rodriguez et al., 2013; Cortes-Tolalpa et al., 2014; Rodriguez et al., 2014; Martínez et al., 2015; Lee et al., 2018). Transcriptome analysis was performed to analyze gene expression in shikimate-producing strains under carbon-limited or phosphate-limited conditions (Johansson et al., 2005; Johansson & Liden, 2006). The highest yield of shikimate in *E. coli* thus far was approximately 71 g/l shikimate with a yield of 0.27 mol/mol glucose in a 1-l fed-batch culture (Chandran et al., 2003).

Previously, an engineered *E. coli* strain showing DHS overproduction was reported (Choi et al., 2019). The strain called Inha103 was constructed to overexpress four shikimate biosynthetic genes (*aroG, aroF, aroB*, and *aroD*) to maximize the metabolite flux to DHS, whereas the *tyrR* involved in feedback inhibition by the aromatic amino acids was deleted. In addition, the *ptsG* and *pykA* genes were deleted, and *ppsA* and *galP* genes were overexpressed to enhance glucose uptake as well as PEP accumulation. The redesigned DHS-overproducing *E. coli* strain, Inha 103, grown in an optimized medium, produced approximately 117 g/l of DHS in 7-l fed-batch fermentation, which was the highest level of DHS production ever reported in *E. coli* (Choi et al., 2019). In the present study, additional engineering was performed to overproduce shikimate starting from the DHS-overproducing strain. The further redesigned shikimate-producing *E. coli* strain produced approximately 101 g/l of shikimate in 7-l fed-batch fermentation, which is the highest level of shikimate production from *E. coli* reported to date. These results suggest that the artificial cell factory design for the shikimate-overproducing strain would be valuable for constructing a microorganism-based high-producing strain for aromatic compounds with industrial value.

## Materials and Methods

### Bacterial Strains, Media, and Culture Conditions

Table 1 lists the bacterial strains and plasmids used in this study. *E. coli* DH5ɑ was used as the cloning host, and *E. coli* K12 & AB2834 were used as the parent strains (Choi et al., 2019). *E. coli* strains for genetic manipulations were grown in Luria-Bertani (LB) medium at 37°C or 30°C. For metabolites production in small-scale culture, a single colony was inoculated in LB medium at 30°C for 15 h, and a secondary culture was inoculated with 1% (v/v) in the same medium at 30°C for 6 h. The miniature culture was grown using 1.3 ml of *E. coli* production medium (EPM) in a 24-well cell culture plate, at 220 rpm under 30°C, for 4 days. The composition of the EPM for shikimate production was: glucose (5 g/l), glycerol (10 g/l), yeast extract (2.5 g/l), tryptone (2.5 g/l), KH_2_PO_4_ (7.5 g/l), MgSO_4_ (0.5 g/l), (NH_4_)_2_SO_4_ (3.5 g/l), NH_4_Cl (2.7 g/l), Na_2_SO_4_ (0.7 g/l), Na_2_HPO_4_•12H_2_O (9 g/l), and a trace metal solution (1 ml/l). The stock trace metal solution contained FeSO_4_•7H_2_O (10 g/l), CaCl_2_•2H_2_O (2 g/l), ZnSO_4_•7H_2_O (2.2 g/l), MnSO_4_•4H_2_O (0.5 g/l), CuSO_4_•5H_2_O (1 g/l), (NH_4_)_6_Mo_7_O_24_•4H_2_O (0.1 g/l), and Na_2_B_4_O_7_•10H_2_O (0.02 g/l).

**Table 1.** Strains and Plasmid Used in This Study

Strains	Relevant characteristics	References
***Escherichia coli* AB2834**	K12 ∆*aroE*	Yale University
***E. coli* Inha103**	AB2834 *∆tyrR ∆ptsG ∆pykA ∆lacI:: Plac_aroB_aroD_Plac_aroG_aroF_Plac_ppsA_galP*	(Choi et al., 2019)
**Inha 201**	AB2834 *∆aroL ∆aroK*	This study
**Inha 202**	AB2834 *∆tyrR ∆ptsG ∆pykA ∆aroL ∆aroK*	This study
**Inha 203**	AB2834 *∆tyrR ∆ptsG ∆pykA ∆lacI:: Plac_aroB_aroD_Plac_aroG_aroF_Plac_ppsA_galP ∆aroL ∆aroK*	This study
**Inha 204**	AB2834 *∆tyrR ∆ptsG ∆pykA ∆lacI:: Plac_aroB_aroD_Plac_aroG_aroF_Plac_ppsA_galP ∆aroL ∆aroK ∆ydiB*	This study
**Inha 205**	AB2834 *∆tyrR ∆ptsG ∆pykA ∆lacI:: Plac_aroB_aroD_Plac_aroG_aroF_Plac_ppsA_galP ∆aroL ∆aroK ∆shiA*	This study
**Inha 206**	AB2834 *∆tyrR ∆ptsG ∆pykA ∆lacI:: Plac_aroB_aroD_Plac_aroG_aroF_Plac_ppsA_galP ∆aroL ∆aroK ∆ydiN*	This study
**Inha 207**	AB2834 *∆tyrR ∆ptsG ∆pykA ∆lacI:: Plac_aroB_aroD_Plac_aroG_aroF_Plac_ppsA_galP ∆aroL ∆aroK ∆shiA ∆ydiN*	This study
**Inha 208**	AB2834 *∆tyrR ∆ptsG ∆pykA ∆lacI:: Plac_aroB_aroD_Plac_aroG_aroF_Plac_ppsA_galP ∆aroL ∆aroK ∆shiA ∆pta-ackA*	This study
**Inha 209**	AB2834 *∆tyrR ∆ptsG ∆pykA ∆lacI:: Plac_aroB_aroD_Plac_aroG_aroF_Plac_ppsA_galP ∆aroL ∆aroK ∆shiA ∆aroE_AB2834_::aroE_K12_*	This study
**Inha 211**	Inha 201/PoppA-aroE	This study
**Inha 212**	Inha 202/PoppA-aroE	This study
**Inha 213**	Inha 203/PoppA-aroE	This study
**Inha 214**	Inha 204/PoppA-aroE	This study
**Inha 215**	Inha 205/PoppA-aroE	This study
**Inha 216**	Inha 206/PoppA-aroE	This study
**Inha 217**	Inha 203/pPoppA-aroE-ydiB	This study
**Inha 218**	Inha 203/PoppA-aroE-tktA	This study
**Inha 219**	Inha 205/PoppA-aroE-tktA	This study
**Inha 220**	Inha 206/PoppA-aroE-tktA	This study
**Inha 221**	Inha 207/PoppA-aroE-tktA	This study
**Inha 222**	Inha 208/PoppA-aroE-tktA	This study
**Inha 223**	Inha 209/PoppA-aroE	This study
**Inha 224**	Inha 209/PoppA-aroE-tktA	This study
**Plasmids**	**Relevant characteristics**	**References**
**pMESK4**	pUC18 modification vector including *oppA* promoter *asbF^Eopt^- aroY^Eopt^- catA^Eopt^*	2019,
**pPoppA-aroE**	pMESK4 modification vector including *oppA* promoter & *aroE_K12_*	This study
**pPoppA-aroE-ydiB**	pMESK1 modification vector including *oppA* promoter & *aroE_K12_*& *ydiB*	This study
**pPoppA-aroE-tktA**	pMESK1 modification vector including *oppA* promoter & *aroE_K12_* & *tktA*	This study
**pCas**	*repA101*(Ts) *kan Pcas-cas9 ParaB-Red lacI*q *Ptrc*-sgRNA-*pMB1*	Addgene, (Jiang et al., 2015)
**pTargetF**	*pMB1 aadA* sgRNA-*cadA*	Addgene, (Jiang et al., 2015)
**pTarget-ydiB**	pTargetF containing sgRNA of *ydiB* and its homologous arms	This study
**pTarget-shiA**	pTargetF containing sgRNA of *shiA* and its homologous arms	This study
**pTarget-ydiN**	pTargetF containing sgRNA of *ydiN* and its homologous arms	This study
**pTarget-pta**	pTargetF containing sgRNA of *pta-ackA* and its homologous arms	This study
**pTarget-aroE**	pTargetF containing sgRNA of *aroE* and its homologous arms	This study
**pTarget-aroE-ins**	pTargetF containing sgRNA of disrupted *aroE* fragment and *aroE_K12_*gene	This study
**pKOV**	The suicide vector containing the *Bacillus subtilis sacB* gene and temperature sensitive pSC101 replication origin	Addgene
**pKOV-aroK**	pKOV containing a PCR fragment for disruption of the *aroK* gene	This study
**pKOV-aroL**	pKOV containing a PCR fragment for disruption of the *aroL* gene	This study

### Construction of Plasmids and Strains

All constructed plasmids are listed in Table 1, and all primer pairs used in this study are shown in Supplementary Table 1. The suicide vector pKOV (Addgene, USA), which has the *sacB* gene for providing a markerless system, was used to disrupt the *aroL* and *aroK* gene.

The CRISPR/cas system was used to disrupt *ydiB, shiA, ydiN, pta-ackA*, and *aroE* genes. Plasmids of pCas and pTargetF were purchased from Addgene (http://www.addgene.org/). CHOPCHOP (https://chopchop.cbu.uib.no/) was used to target the sgRNA of the gene loci of interest. The homologous DNA fragments and sgRNA were PCR-amplified with primer sets, such as ydiB1-ydiB5, shiA1-shiA5, ydiN1-ydiN5, pta1-pta5, and aroE1-aroE5, which were then inserted into the pTargetF vector, generating pTarget-ydiB, pTarget-shiA, pTarget-ydiN, pTarget-pta, and pTarget-aroE, respectively. The experimental method for gene editing is reported elsewhere. To insert the *aroE_K12_* gene in the *aroE* loci, pTarget-aroE-ins was constructed for targeting the *aroE* disrupted gene loci and used to replace the disrupted *aroE* to the whole *aroE_K12_* gene.

For gene overexpression, the plasmids of pPoppA-aroE, pPoppA-aroE-tktA, and pPoppA-aroE-ydiB were constructed. The pPoppA plasmid was amplified from pMESK4, which contained the *oppA* promoter and used as a backbone. The genes of interest were amplified and cloned into the pPoppA plasmid digested with *Nde*I for *aroE* and *Hind*III for *tktA* and *ydiB*.

### Fed-batch Fermentation

The first growth culture was carried out in a conical tube containing 5 ml of LBG medium (30°C and 200 rpm). After 12 h of culture, a 250-ml baffled flask containing 20 ml of culture medium was inoculated with 0.2 ml of the culture broth and cultured at 30°C and 200 rpm in an incubator. After 6 h, a 7-l fermenter (1% v/v inoculum) was inoculated with the second culture broth. The production culture was carried out using the PB4-md5 medium in a 7-l fermenter (2 l working volume). The PB4-md5 medium included the following: 30 g/l glucose, 10 g/l glycerol, 15.75 g/l yeast extract, 21.375 g/l tryptone, 5.25 g/l KH_2_PO_4_, 1 g/l MgSO_4_∙7H_2_O, 0.8 g/l citric acid, 1 ml/l trace metals, and 200 µg/l thiamine hydrochloride. The feeding medium included 600 g/l glucose, 100 g/l yeast extract, 20 g/l MgSO_4_∙7H_2_O, and 5 ml/l trace metal. Phosphate was not added to the feeding medium to allow for the regulation of cell growth. The pH was 7.0 in each culture (10N NaOH, 3M HCl), and the DO level was maintained above 30% by controlling the agitation, aeration, and feeding rates. The configuration of the fermenter was as follows: two impellers with a turbine and marine impeller, ring-type sparger with 12 holes, top-driven, and 160 mm tank diameter. The feeding medium was supplied using a peristaltic pump when glucose was depleted (Choi et al., 2019).

### Shikimate and DHS Analyses

The *E. coli* cells were removed from the cultures by centrifugation, and the culture broth was purified using a membrane filter. The metabolites were separated by high-performance liquid chromatography (HPLC) using an Aminex HPX-87H column. The mobile phase was 2.5 mM H_2_SO_4_, and a flow rate of 0.5 ml/min was used for the metabolites. The column was heated to 50°C to detect the metabolites. Shikimate and DHS were analyzed at wavelengths of 215 nm and 236 nm, respectively. Organic acids, including succinic acid, acetic acid, formic acid, and lactic acid, were detected at 210 nm.

### Metabolome Analysis and Profiling

For metabolite analysis, the Inha 212 (Supplementary Fig. S4A) and Inha 219 (Supplementary Fig. S4B) strains were cultured in 3 l of PB4-md5 media using a 7 l fermenter. The growth curves were generated, and each strain was sampled at 8 h, 13 h, 24 h, 48 h, and 72 h. The cells collected at each time point were washed twice with H_2_O and frozen. Metamass Inc., Korea, performed metabolite analysis. The sample-solvent mixtures were centrifuged (at 5000 rpm for 10 minutes at 4°C), and the solvent layers were then collected and dried using a speed vacuum concentrator (Hanil Scientific, Seoul, Korea). The weights of the dried solvent layers were then determined by gas chromatography–time-of-flight mass spectrometry (GC-TOF-MS) analysis. The raw data sets from GC-TOF-MS were converted to the Net CDF format (*.cdf). Principal component analysis (PCA), partial least squares discriminant analysis (PLS-DA), and orthogonal PLS-DA (OPLS-DA) modeling were then performed to compare each data set.

## Results

### Redesign of Shikimate Biosynthetic Pathway in *Escherichia coli*

To construct a strain capable of accumulating shikimate, an attempt was made to redesign a strain using the previously constructed DHS-overproducing *E. coli* strain Inha 103 (Choi et al., 2019). As shown in Fig. 1, DHS is another key metabolite present as an immediate precursor of shikimate in the pathway. Inha 103 was constructed rationally to maximize DHS accumulation through the DHS biosynthetic pathway engineering. The *tyrR* was deleted to inhibit the feedback inhibition of the DAPH synthase isozymes encoded by *aroG* and *aroF* by tyrosine. Phosphoenolpyruvate phosphotransferase (encoded by *ptsG*), which plays a role in converting PEP to pyruvate, was deleted. Pyruvate kinase 2 (encoded by *pykA*) was also deleted to inhibit the conversion of PEP to pyruvate through glycolysis. Several key DHS biosynthetic pathway genes, including *aroG, aroF, aroB*, and *aroD*, were overexpressed through chromosomal integration into the *lacI* location. The D-galactose transporter (encoded by *galP*) and PEP synthase (encoded by *ppsA*) genes were also inserted to maximize DHS overproduction.

**Fig. 2.**
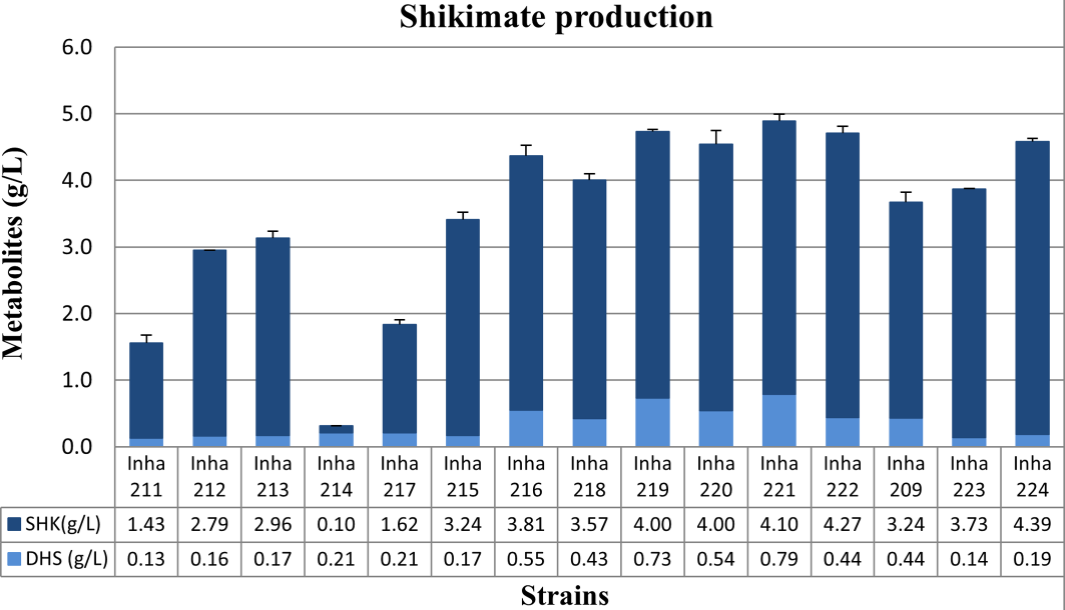
Shikimate production yield in shikimate-producing *Escherichia coli* strains. Data represent averages and standard deviations from two independent experiments.

To prevent the metabolic processing of shikimate to a next step metabolite, shikimate 3-phosphate, the *aroK* and *aroL* genes encoding shikimate kinase were first disrupted in the Inha103 strain (named Inha 203; Table 1). The inactive form of *aroE* in the Inha 203 strain was restored by cloning a wild-type functional *aroE* and expressing it under the strong promoter *oppA* in the Inha 203 strain (called Inha 213). The Inha 213 strain (AB2834 *∆tyrR ∆ptsG ∆pykA ∆lacI::Plac_aroB_aroD_Plac_aroG_aroF_Plac_ppsA_galP ∆aroL ∆aroK/PoppA-aroE*) accumulated approximately 2.96 g/l of shikimate. In contrast, the control strains, Inha 211 (AB2834 *∆aroL ∆aroK/PoppA-aroE*) and Inha 212 (AB2834 *∆tyrR ∆ptsG ∆pykA ∆aroL ∆aroK/PoppA-aroE*), produced 1.43 g/l and 2.79, respectively, under the same culture conditions (Fig. 2; Table 1).

In addition to the NADP-specific AroE, there is an additional AroE-like shikimate dehydrogenase called YdiB. YdiB plays a role as a dual-specificity quinate/shikimate dehydrogenase that can use either NAD or NADP as cofactors (Michel et al., 2003; Garcia et al., 2017). The expression of *ydiB* was increased significantly under carbon-limiting conditions, and it is involved in the catabolism of shikimate (Johansson & Liden, 2006). To check the putative function of YdiB in shikimate production, a strain called Inha 214 was constructed, in which the *ydiB* gene was deleted. Although the growth pattern was compatible with other strains, the Inha 214 strain failed to produce shikimate under the same culture conditions (Fig. 2, Supplementary Fig. S1). Interestingly, the strain Inha 217, overexpressing the *ydiB* gene, accumulated approximately 1.62 g/l of shikimate, suggesting that the AroE and YdiB enzymes might act competitively to influence the conversion of DHS to shikimate. The role of shikimate dehydrogenase by AroE is believed to be the most relevant factor in these strains and culture conditions.

### Modification of *shiA, ydiN, tktA, and pta-ackA* for Shikimate Overproduction

*shiA*, one of two putative transporter genes in *E. coli*, plays a role in importing shikimate and DHS into the cell. The other transporter is encoded by *ydiN*, even though its precise function is unknown (Knop et al., 2001; Martinez et al., 2015). Shikimate has been proposed to be carried in-and-out of the cell by ShiA and YdiN depending on the C-limitation and P-limitation conditions (Kanehisa & Goto, 2000; Johansson & Liden, 2006). The fed-batch culture showed that the production of shikimate decreased toward the later stages of culture (Supplementary Fig. S2), suggesting that overproduced shikimate might be reintroduced into the cell for recycling. To determine if the deletion of *shiA* or *ydiN* affected the production of shikimate, these two genes were removed from Inha 213 to construct the Inha 215 and Inha 216 strains, respectively. The production yields of shikimate in Inha 215 and Inha 216 strains were approximately 1.1 and 1.3 times higher than that of the parental strain Inha 213, respectively (Fig. 2).

Many studies have reported that the overexpression of the *tktA* increased the production of metabolites in the shikimate pathway through the accumulation of E4P and PEP, which are important precursors of shikimate biosynthesis; Knop et al., 2001; Escalante et al., 2010). Therefore, the expression vector containing both *aroE* and *tktA* was constructed under a strong OppA promoter (Choi et al., 2019), which was then expressed in several shikimate-producing *E. coli* strains stated above (Table 1). When *tktA* was overexpressed in the *shiA*-deleted strain (Inha 219), the shikimate yield was 4.0 g/l, which was 1.4 and 1.1 times higher than that of Inha 212 and Inha 213, respectively. Moreover, the Inha 221 strain, in which the *tktA* was overexpressed in the *shiA* and *ydiN* double knock-out strain, produced 4.10 g/l shikimate, showing the highest production of shikimate among the strains tested. Interestingly, when the Inha 221 strain was cultured in a 7-l fed-batch fermentation, the level of shikimate production was not higher than that from Inha 219 (see below, Supplementary Fig. S3).

Two enzymes, phosphate acetyltransferase (encoded by *pta*) and acetate kinase (encoded by *ackA*) are believed to be involved in acetate formation (Fig. 1). Comparative metabolite analysis showed that a small amount of acetate accumulated as a by-product in the Inha 219 strain at 72 h in the late culture period (Supplementary Fig. S5). Although these two genes were deleted from the chromosome (called Inha 222), the shikimate production titer was not improved significantly (data not shown).

### Fed-batch Fermentation for the *aroE*-Overexpressing Shikimate Producer

AroE plays a critical role in the metabolic processing of DHS to shikimate. Hence, an inactive chromosomal *aroE* was replaced with an *E. coli* K12-derived active *aroE* in the chromosome of Inha 209 along with a plasmid-driven functional *aroE* (named Inha 223). Inha 209 and Inha 223 produced 3.24 and 3.73 g/l of shikimate, respectively, suggesting that the simultaneous overexpression of *aroE* from the chromosome and the plasmid is beneficial for shikimate overproduction. Moreover, the Inha 224 strain, overexpressing both *aroE* and *tktA* genes in the plasmid, showed a further increase in shikimate production to 4.39 g/l, which is the highest production level among the strains tested in a miniature culture system.

**Fig. 3.**
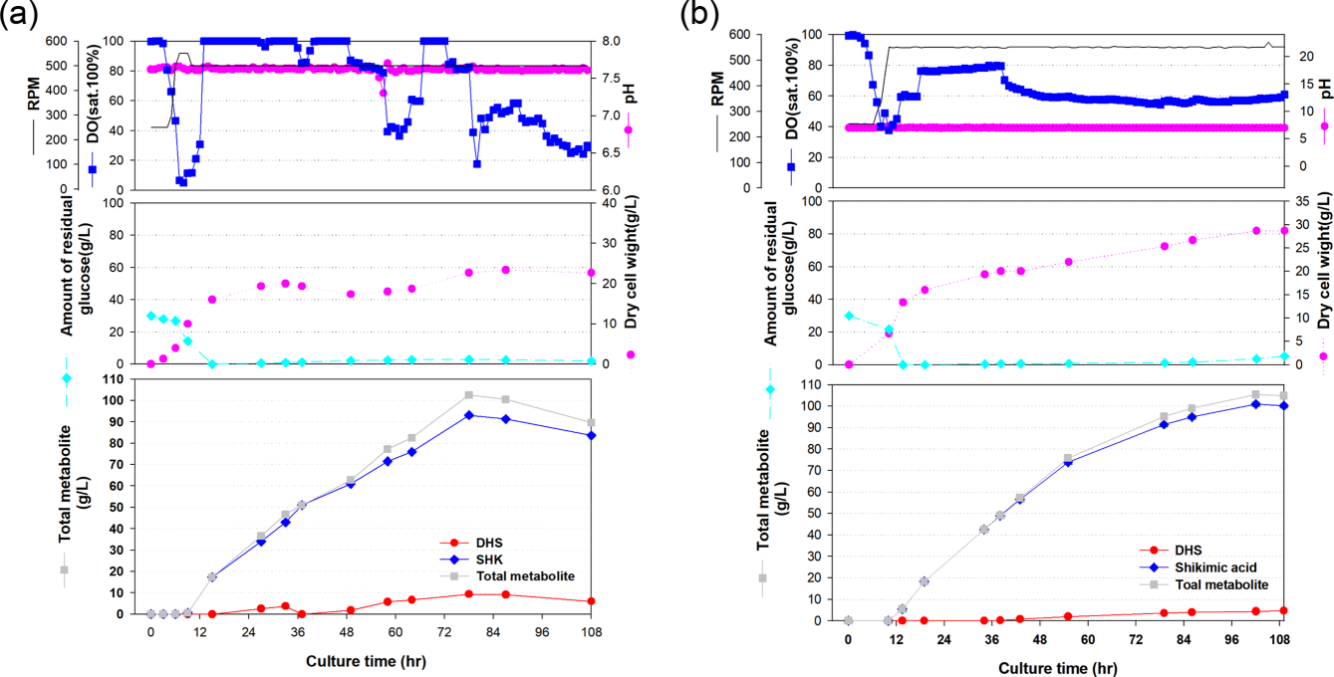
Time-course profiles of cell growth (DCW), glucose, organic acid, and metabolite production by Inha 219 (a) and Inha 224 (b) strains in the 7-l fermenter. (a) The feeding medium was sequentially injected at culture periods of 11, 41, and 85 h, at a rate of 0.1701, 0.2268, and 0.2646 ml/min, respectively. (b) The feeding medium was sequentially injected at culture periods of 13.5 and 39.5 h at a rate of 0.1701 and 0.2268 ml/min, respectively.

Accordingly, 7-l fed-batch fermentation was performed using the highest shikimate-producing strain of Inha 224. It exhibited the maximum cell dry weight of approximately 28 g/l and a shikimate level of approximately 101 g/l at 102 h of culture (Fig. 3b). The Inha 224 strain showed a slightly higher DCW than the Inha 219 strain, but the maximum yield of shikimate in Inha 224 was increased by approximately 8%. The specific production yield (Y_p/s_) was 0.33 g shikimate/g glucose for the strain Inha 213, 0.34 g shikimate/g glucose for the Inha 213 applying an optimized process, 0.48 g shikimate/g glucose for the Inha 219 strain, and 0.47g shikimate/g glucose for the Inha 224 strain (Table 2). Comparing to the Inha 213 strain, the shikimate yield from the Inha 224 strain was improved 1.42 times to 101 g/l, which is the highest level of shikimate production reported for *E. coli*.

**Table 2.** *P_f_*, Maximum Shikimate Production (g IA/l); *X_f_*, Maximum Dry Cell Weight (g DCW/L); *S_f_*, Final Residual Glucose Concentration (g Glucose/l); *Q_p_*, Average Volumetric DHS Production Rate (g Shikimate/l/h); *q_p_*, Average Specific Shikimate Production Rate (g Shikimate/g DCW/h); *Y_p/x_*, Specific Shikimate Production (g Shikimate/g DCW); *Y_p/s_*, Shikimate Production Yield Based on Glucose (g Shikimate/g Glucose); *Y_x/s_*, DCW Yield Based on Glucose (g DCW/g Glucose)

	P_f_	X_f_	S_f_	Q_p_	q_p_	Y_p/x_	Y_p/s_	Y_x/s_
**Inha 213**	67	38	1	0.67	0.017	1.7	0.33	0.10
**Inha 219**	93	22	2	1.27	0.057	4.2	0.48	0.11
**Inha 224**	101	28	2	1.03	0.037	3.6	0.47	0.13

## Discussion

In this study, shikimate-overproducing *E. coli* was generated successfully by genetic and metabolic engineering approaches. A strain was constructed to produce shikimate based on a previously designed DHS-overproducing strain that strengthened the biosynthetic pathway of shikimate, resisted feedback inhibition, increased glucose uptake, and induced major precursor accumulation. Furthermore, the shikimate kinase genes, *aroL* and *aroK*, were deleted, and the functional *aroE* gene encoding shikimate dehydrogenase to convert from DHS to shikimate was inserted. YdiB, which encodes shikimate dehydrogenase, such as AroE in *E. coli*, has dual specificity of quinate/shikimate dehydrogenase using NAD or NADP as a cofactor, unlike the NADP-specific AroE (Michel et al., 2003). One study reported that the molar yield of quinate, a by-product of shikimate biosynthesis, decreased when YdiB was inactivated in the PTS-strain of *E. coli*, and shikimate production decreased slightly. In contrast, when YdiB was overexpressed, quinate production increased 150% mol/mol compared to shikimate (Garcia et al., 2017). In the present study, shikimate was barely produced when the *ydiB* gene was deleted. When *ydiB* was overexpressed, the shikimate productivity was 55% lower than that of the parental strain. Johansson and Lidén (Johansson & Liden, 2006) reported the overexpression of *ydiB* with respect to *aroE* in a global microarray-based transcriptomic analysis. They suggested that YdiB, rather than AroE, catalyzes the oxidation of shikimate to DHS in the shikimate-producing *E. coli* W3110.shik strain grown under carbon-limiting and nonlimiting conditions. Therefore, shikimate production might be affected by the competitive shikimate dehydrogenase role of YdiB and AroE, depending on the culture conditions. This suggests that the NADP-specific AroE should act as the main shikimate dehydrogenase. On the other hand, this effect is different in the state of limited energy and carbon in the cell depending on the expression of YdiB, which requires a broad cofactor. Confirming the production of other intermediate products, such as DHQ and quinate, during the biosynthesis process is important from various viewpoints, such as the flow of energy in the cell or the limits of carbon or nitrogen in the medium.

In this study, an additional increase in the shikimate level was confirmed by deleting ShiA, a shikimate transporter. The Inha 219 strain, which simultaneously overexpressed *tktA* and *aroE*, showed higher productivity of shikimate than when *shiA* was deleted alone. When shikimate enters the cell to be used as a carbon source, it causes catabolite repression and induces the accumulation of intermediates, such as quinate and gallate (Knop et al., 2001; Chandran et al., 2003; Kramer et al., 2003). Therefore, the strategy of inactivation of the shikimate transporter was used to reduce the accumulation of by-products. In addition, the transport of shikimate by ShiA can be bidirectional, and that shikimate can be transported by other transporters, such as YdiN (Johansson et al., 2005). In this study, the shikimate production efficiency could be improved by preventing the re-inflow of shikimate from outside of the cells in small-scale cultivation through the deletion of *shiA* or *ydiN*, or both, and allowing the biosynthesis of shikimate from glucose. In addition, a strategy to improve the E4P pool for the synthesis of DAHP through overexpression of the *tktA* gene encoding transketolase I has proven its effectiveness through many studies (Draths et al., 1999; Chandran et al., 2003; Escalante et al., 2010; Rodriguez et al., 2013). In the present study, the resulting strain Inha 219, which has a *shiA*-deletion and *tktA*-overexpression, produced 93 g/l of shikimate at a yield of 0.48 g/g after 78 h in 7-l fed-batch fermentation with the modified medium. In addition, the active AroE expressing Inha 224 strain produced 101 g/l of shikimate at a yield of 0.48 g/g after 102 h in 7-l fed-batch fermentation. To the best of the authors’ knowledge, this is the highest shikimate titer and yield obtained using a microbial catalyst reported so far. The transport of shikimate into the cell may be repressed by the glucose catabolism, resulting in fewer by-products formed under C-rich conditions (Knop et al., 2001). Such a remarkable production yield was achieved by applying several metabolic engineering strategies for shikimate production in *E. coli* strains. The DCW of Inha 219 was lower than that of Inha 224, but the shikimate production yield was high, suggesting that there is a competitive relationship between cell growth and shikimate production. Although shikimate is a primary metabolite, its production in engineered strains exhibited typical secondary metabolite patterns. In aromatic amino acid production processes, as shown previously in the production of DHS and MA, *E. coli* and *Corynebacterium glutamicum* have characteristic metabolites that suppress the cell density at certain concentrations (Cheng et al., 2012; Rodriguez et al., 2014; Lee et al., 2018).

Studies on the production of shikimate from various microorganisms other than *E. coli* have also been reported. Engineered *C. glutamicum* strains show the highest shikimate productivity after *E. coli*. After constructing the genetic modules of the *aroG* (NCgl2098), *aroB* (NCgl1559), *aroD* (NCgl0408), and *aroE* (NCgl1567) genes based on the selected RBS libraries, *C. glutamicum* RES167*ΔaroK* carrying an efficient genetic module produced 7.4 and 11.3 g/l of shikimate during 5-l batch and fed-batch fermentation, respectively (Zhang et al., 2015). The CRISPRi system has been used to regulate *C. glutamicum* gene expression at the transcriptional level (Zhang et al., 2016). The titers of shikimate increased from 115% to 7.76 g/l in 250 ml flasks and 23.8 g/l in a 5-l fermenter. The highest productivity from the SKM7 strain (*C. glutamicum ∆aroK ∆qsuB ∆qsuD ∆glks: glk1 glk2 ∆ppgk ∆ptsH ∆hpdA* overexpressed *iolT1 glks gapA tkt tal aroGFBR, aroB, aroD, aroE*) was 97.1 g/l shikimate at a 30.1% yield (mol/mol) after 80 h in fed-batch fermentation experiments in rich medium without the switch to a minimal medium (Kogure et al., 2016). The highest yield of 141 g/l shikimate (51% mol/mol) was obtained by the growth-arrested cell reaction method.

Genetically manipulated *Bacillus subtilis* (Ghosh & Banerjee, 2015; Iomantas et al., 2002) and *Citrobacter freundii* strains (Shirai et al., 2001) have been exploited successfully to produce shikimate, but the titers did not exceed 20 g/l. A shikimate kinase (*aroI*)-inactivated *B. subtilis* was shown to produce 8.5 g/l shikimate together with 9.5 g/l of DHS (Ghosh & Banerjee, 2015). Many studies also have produced various aromatics and derived compounds using *Saccharomyces cerevisiae* (Averesch & Kromer, 2018). Gao et al. reported that the development of a new platform based on more efficient xylose utilization makes *Scheffersomyces stipites* particularly suited to produce the shikimate group of compounds. Shikimate was produced at 3.11 g/l, representing the highest level among the products of the shikimate pathway in yeasts (Gao et al., 2017).

In summary, this paper reported the construction of *E. coli* strains capable of producing shikimate at high concentrations from D-glucose. To accumulate high concentrations of shikimate, *tyrR, ptsG*, and *pykA* genes were deleted sequentially from *E. coli* AB2834, in which the *aroE* gene was mutated to prevent the conversion of DHS to shikimate. Extra copies of *aroB, aroD, galP, ppsA, aroG*, and *aroF* involved in shikimate biosynthesis were also inserted to maximize shikimate accumulation. To convert DHS to shikimate, the *aroE* gene was introduced so that it could be expressed under a strong *oppA* promoter. A controlled fed-batch operation was performed with a statistically optimized production medium in a 7-l bioreactor. The redesigned *E. coli* strain produced 101 g/l shikimate and 9.2 g/l DHS with almost no accumulation of metabolic intermediates, such as acetate and quinate. This study demonstrates the potential of *E. coli* to produce high levels of intermediate metabolites of aromatic pathways and a rational cell factory design approach to manufacture valuable aromatic compounds.

## Author Contributions

S.J.L. and E.S.K. designed the research. H.N.L., S.Y.S., J.H.P., H.J.K., and S.S.C. performed the experiments, as well as data collection and analysis. H.N.L., J.H.P., and E.P. performed genetic engineering. S.Y.S. performed medium optimization and fermentation. H.N.L., S.Y.S., and S.S.C. wrote the article. S.S.C., S.J.L., and E.S.K. provided the improvement of the manuscript.

## Funding

This work was carried out with the support of “Cooperative Research Program for Agriculture Science and Technology Development (Project No. PJ01318701),” Rural Development Administration, Republic of Korea.

## Conflict of Interest

The authors declare no conflict of interest.

## Supplementary Material

kuab043_Supplemental_File
