# Supplementary material for: Artificial cell factory design for shikimate production in Escherichia coli
Source: J Ind Microbiol Biotechnol. 2021 Jul 6;48(9-10):kuab043. doi: 10.1093/jimb/kuab043 (PMC8788726; doi:10.1093/jimb/kuab043)
Supplement: kuab043_Supplemental_File [file kuab043_supplemental_file.docx]

**Artificial Cell Factory Design for Shikimate Production in *Escherichia coli***

**Han-Na Lee^1,2^, Seung-Yeul Seo^2^, Hey-Jin Kim^1^, Ji-Hoon Park^1^, Eunhwi Park^1^, Si-Sun Choi^1^, Sang Joung Lee^2^, and Eung-Soo Kim^1,^***

^1^Department of Biological Sciences and Bioengineering, Inha University, Incheon 22212, Republic of Korea

^2^ STR Biotech Co., Ltd., Bioplaza 4-3, 56, Soyanggang-ro, Chuncheon-si, Gangwon-do 24232, Republic of Korea

^*^Corresponding author: Eung-Soo Kim

*Department of Biological Engineering, Inha University, Incheon 22212, Korea*

Tel: 82-32-860-8318, Fax: 82-32-872-4046, E-mail: eungsoo@inha.ac.kr

**Supplemental Figure 1.** Cell proliferation comparison results between Inha 213 and Inha 214 strains. The measured OD_600_ value after the completion of the small-scale culture was shown. Data represent independent cell cultivation.


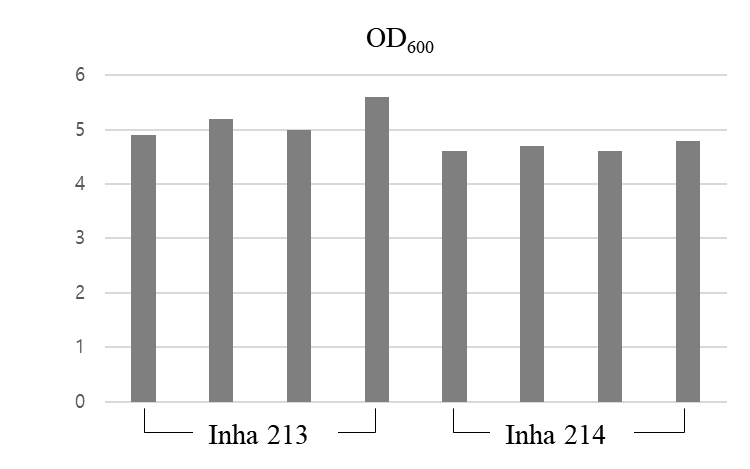


**Supplemental Figure 2.** Time-course profiles of cell growth (DCW), glucose, organic acid, and metabolite production by Inha 213 in the 7-L fermenter. The feeding medium was sequentially injected at culture periods of 17, 42, 45, 69, 76, and 90 h, at a rate of 0.1701, 0.2646, 0.1701, 0.3402, 0.2835 and 0.2268 mL/min, respectively.

**Supplemental Figure 3.** Time-course profiles of cell growth (DCW), glucose, organic acid, and metabolite production by Inha 221 in the 7-L fermenter. The feeding medium was sequentially injected at culture periods of 12, 42, and 81 h, at a rate of 0.189, 0.2268 and 0.2646 mL/min, respectively.

**Supplemental Figure 4.** Comparative fed-batch fermentation of Inha 212 (A) and Inha 219 (B) strains in 7-L fermenter. (A) The feeding medium was sequentially injected at culture periods of 9h, at a rate of 0.1701, mL/min, respectively. (B) The feeding medium was sequentially injected at culture periods of 9, and 48 h, at a rate of 0.1701, and 0.2268 mL/min, respectively. Cells were collected at 8 h, 13 h, 24 h, 48 h, and 72 h.

**
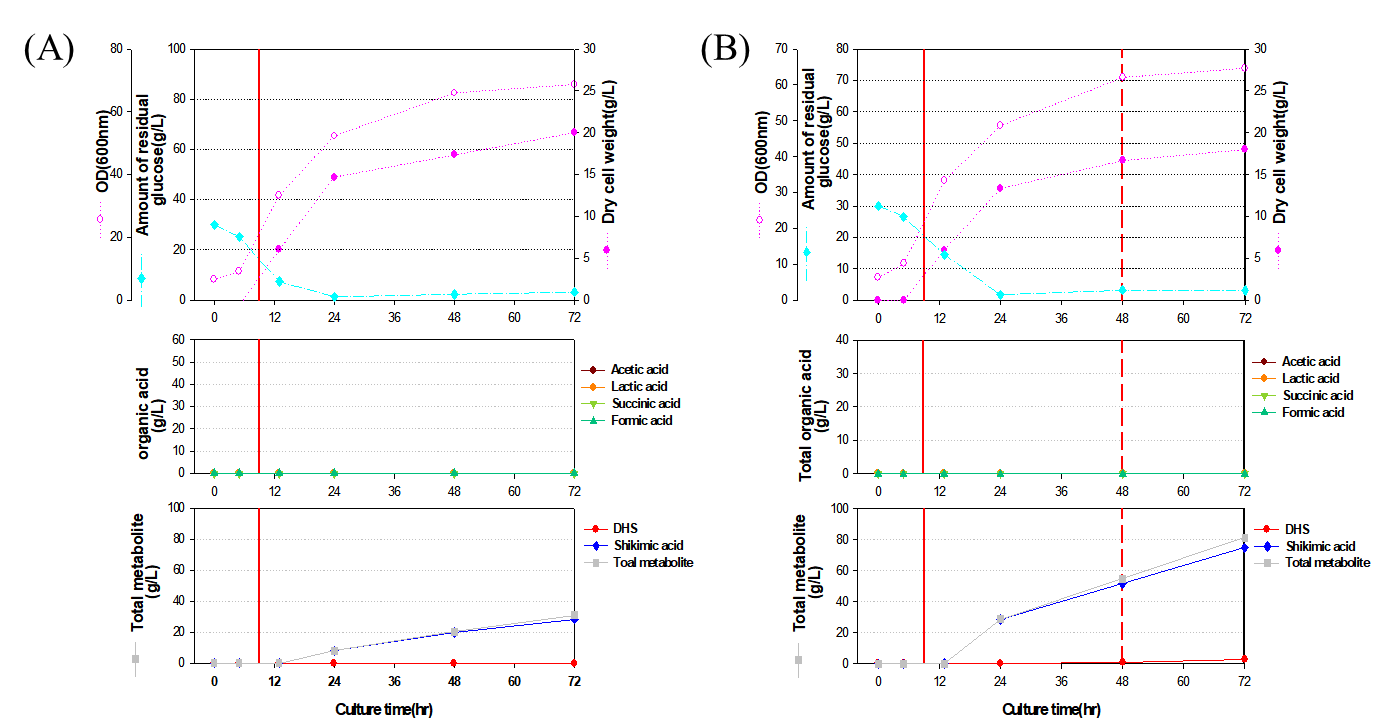
**

**Supplemental Figure 5.** Metabolite analysis between Inha 212 strain (blue line) and Inha 219 strain (orange line).

**
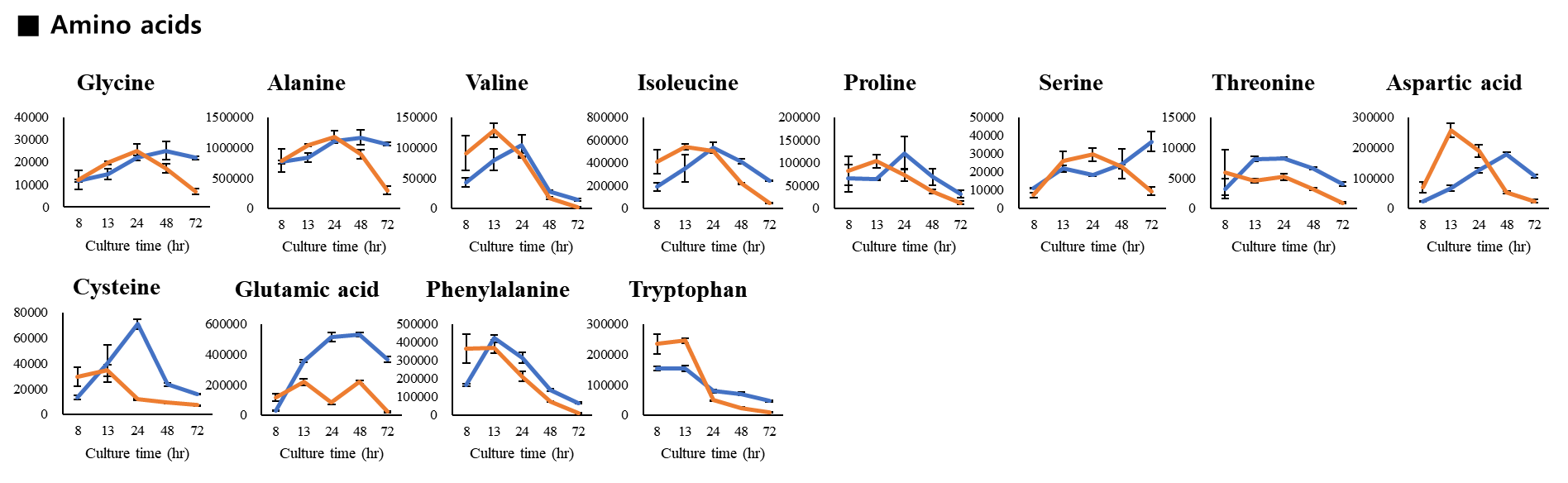
**

**
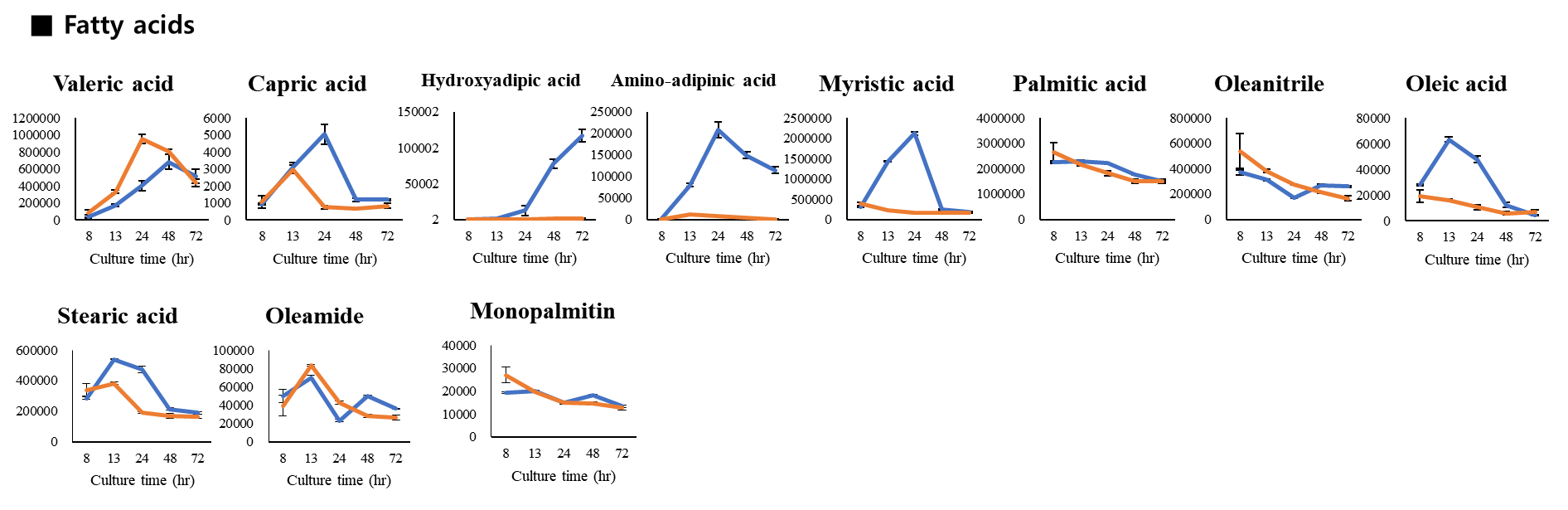
**

**
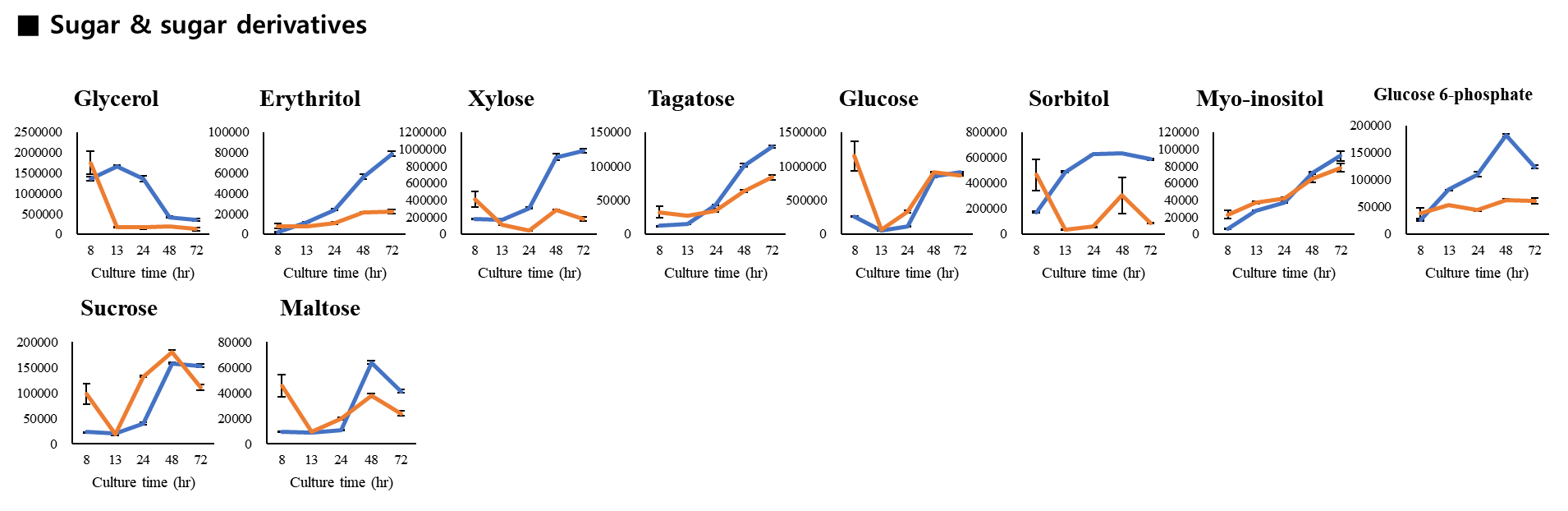
**

**Supplemental Table 1.** Primer list used in this study.

| **Primer name** | **Sequence (5’🡪 3’)** | **Target** |
| --- | --- | --- |
| K1 | tttcgcacctgggatccaatacgcctgcgcggacattt | *aroK* gene disruption |
| K2 | CGACTCTAGAGGATCCGTTGGCCAATGAACGCAATCC |  |
| K3 | TCCTCTAGAGTCGACTTGAGTTGTTGAGCTAACTGG |  |
| K4 | CCATTCTCCGGTCGATCTGCTGGCCTCACATCTTC |  |
| L1 | TTCGCACCTGGGATCGACGCGTGTCCCAATGTAAT | *aroL* gene disruption |
| L2 | CACCTGGCTGGGTTCACGGTTAAGCGAATCGGCAA |  |
| L3 | GAACCCAGCCAGGTGATTTC |  |
| L4 | CGACTCTAGAGGATCTGAAGCACCACTGCTGACAC |  |
| ydiB1 | GTCCTAGGTATAATACTAGTctgcgtggctataacaccgaGTTTTAGAGCTAGAAATAGC | pTarget-ydiB |
| ydiB2 | ttctctagagtcgacaacagcaaaccgttagtctg |  |
| ydiB3 | cacgcggtcagcctgttaacctctatgcttaattg |  |
| ydiB4 | aagcatagaggttaacaggctgaccgcgtgcagaa |  |
| ydiB5 | cttctgcaggtcgacccaaatacttcaccagccag |  |
| shiA1 | GTCCTAGGTATAATACTAGTATTCAGGGATTTGCAGTCGGGTTTTAGAGCTAGAAATAGC | pTarget-shiA |
| shiA2 | gacctgcagaagctaatatggatgacaacaaagt |  |
| shiA3 | gtcgacgacggcaccagcgaagctgc |  |
| shiA4 | ggtgccgtcgtcgacaaagacagtcaacgcgctt |  |
| shiA5 | aatagatctaagcttccagttctgttgtcgggaag |  |
| ydiN1 | GTCCTAGGTATAATACTAGTGTGGATGCCCAAATATGCGAGTTTTAGAGCTAGAAATAGC | pTarget-ydiN |
| ydiN2 | ttctctagagtcgacgcaatattcttttcaggtca |  |
| ydiN3 | cgcacactgccagaccgtaggcgaga |  |
| ydiN4 | gtctggcagtgtgcgtttgttattccactgattac |  |
| ydiN5 | cttctgcaggtcgacgccatcatcattaacgatgg |  |
| Pta1 | GTCCTAGGTATAATACTAGTcaaagaaggtacccgtcctgGTTTTAGAGCTAGAAATAGC | pTarget-pta |
| Pta2 | tggtttagccgaatgtttccacct |  |
| Pta3 | gtaaggcagggcgtagaggtaaga |  |
| Pta4 | tacgccctgccttacgtgcgaacgtgctttcgcta |  |
| Pta5 | aatagatctaagcttgctgttcgcgggcaacggtt |  |
| aroE1 | GTCCTAGGTATAATACTAGTTGATGATTTAGGAATGCTGGGTTTTAGAGCTAGAAATAGC | pTarget-aroE(del) |
| aroE2 | cagaagcttagatctagcaaattacgagcagctt |  |
| aroE3 | tatgttacccctgtcgaaacagttc |  |
| aroE4 | atcaggccatccagtttccggaca |  |
| aroE5 | cagggtaatagatctggcaatcgagttttggttgg |  |
| aroE6 | GTCCTAGGTATAATACTAGTgaacgctttcttaggaatgcGTTTTAGAGCTAGAAATAGC | pTarget-aroE-ins |
| aroE7 | cagaagcttagatctatggaaacctatgctgtttt |  |
| aroE8 | cagggtaatagatcttcacgcggacaattcctcct |  |
| ydiB over1 | gtccgcgtgaagctaaaggagttaattatggatg | pPoppA-aroE-ydiB |
| ydiB over2 | tgtcggggcAAgcttcacgcggtcagcctgtcag |  |
| tkt over1 | gtccgcgtgaagcTTaagggcgtgcccttcatcat | pPoppA-aroE-tktA |
| tkt over2 | tgtcggggcAAgctttaattacagcagttcttttg |  |
